# Nutritional research is moving to a whole-diet approach, time for food policy

**DOI:** 10.1186/s12916-021-01984-9

**Published:** 2021-04-30

**Authors:** Briar L. McKenzie, Lindsay M. Jaacks

**Affiliations:** 1 The George Institute for Global Health, University of New South Wales, Sydney, Australia; 2 Global Academy of Agriculture and Food Security, The University of Edinburgh, Midlothian, UK

**Keywords:** Dietary patterns, Dietary intake, Sex differences, Obesity, Food policy, Cardiovascular disease

## Background

Obesity has been implicated in the recent slowing of longstanding declines in cardiovascular disease (CVD) mortality in the UK [1]. Indeed, about a decade ago, overweight and obesity overtook smoking as the leading cause of deaths in England and Scotland [2]. Poor diets underlie these trends, and poor policies underlie poor diets. One potential reason we have not improved population diets is that food policies (or, in most cases, voluntary action by the food industry) have tinkered with ‘nutrients of concern’ (e.g. sodium, sugar, and trans-fat) rather than taking a whole-diet approach. Largely, this reflects nutritional research, wherein the majority of studies have focused on the association of single nutrients or foods with disease risk factors or, occasionally, hard endpoints (e.g. CVD).

## Main text

In a recent study published by *BMC Medicine*, Gao and colleagues take a different approach [3]. They investigate the association of the whole diet, or ‘dietary patterns’, with CVD and death using data from the UK Biobank. Half a million people, aged 40–69 years at baseline, volunteered to participate in the UK Biobank cohort study since 2006. About one in four of these participants has provided detailed information on their dietary intake via two or more 24-h recalls conducted using online software. The authors used reduced rank regression to identify dietary patterns most predictive of nutrients that lead to energy imbalance and obesity [3]. They found that a dietary pattern characterised by high levels of chocolate and confectionary, butter, white bread, and table sugar and preserves, and low levels of fresh fruit, vegetables and whole grains explained 43% of the population’s variation in energy density, free sugars, saturated fat and fibre.

The prevalence of this type of diet is concerning, given that the UK Biobank population is considered a ‘healthy volunteer population’. Only 7% of the cohort was current smokers at baseline, compared to 21% in the general population at a similar time [4]. There were also significant differences in the characteristics of people who had diets that aligned more with this unhealthy pattern. Namely, they were much more likely to be men (63% were men in the highest quintile of the unhealthy dietary pattern versus 29% men in the lowest quintile) [3]. Given that those with diets that aligned more with this unhealthy dietary pattern had an increased risk of CVD and death, these findings may partly explain why ischaemic heart disease remains the leading cause of death amongst men in the UK, whilst in women, dementia and Alzheimer’s disease have been the leading cause of death since 2011 [5].

Efforts are being made to improve diets (or, rather, specific nutrients) in the UK, including effective initiatives to reduce population salt intake [6]. In 2016, a sugar levy was announced placing a higher tax on sugar-sweetened beverages, with a small reduction in sugar sweetened beverage purchases evident a year after implementation [7]. Pressured by the COVID-19 pandemic, and the association between obesity and worse COVID-19 outcomes, the UK government devised an Obesity Strategy [8]. Whilst the strategy largely relies on ‘individual empowerment’, there are some broader policy measures, including (1) to legislate the end of promoting foods high in fat, sugar or salt (e.g. to stop ‘2 for the price of 1’ promotions on certain foods) and (2) to ban the advertising of foods high in fat, sugar or salt on television or online before 9 pm. Although salt was not included in the Gao et al. study [3], many of the foods identified within their harmful dietary pattern will be targeted by these policy measures. The impact of these new initiatives on diets in the UK needs to be monitored and further, stronger legislation adopted in the event that clinically meaningful improvements are not observed.

Poor diets and obesity are the biggest disease risk factors in the UK. There is an urgent need to improve the way and the frequency with which we assess dietary intake, not just for researchers and public health nutritionists, but also general practitioners (GPs). A key component of the UK Obesity Strategy is equipping staff in Primary Care Networks to be ‘healthy weight coaches’ [8]. The strategy highlights the role of GPs in facilitating change in patient smoking behaviour and sees an opportunity in doing the same for poor diets. However, conducting a traditional diet assessment in a 10–15-min GP appointment is not feasible. There is a clear need for re-thinking how we assess diets—and who assesses diets—in the context of this new Obesity Strategy. Support could also feed into monitoring of nutritional adequacy of UK diets more broadly. If GPs start collecting diet information as part of routine assessments, those data could be captured and used to monitor population diets and the association of dietary intake with health outcomes.

## Conclusions

In 1993, Barry Popkin described the theory of the ‘nutrition transition’, including five stages [9]. The fourth stage, the ‘degenerative disease’ stage, is where the UK is now: poor diets leading to obesity and degenerative disease. Thirty years later, the fifth stage remains largely hypothetical: it is a stage wherein the population consumes a ‘healthy’ dietary pattern and witnesses corresponding decreases in noncommunicable diseases. It would be nice to someday do a data-driven dietary pattern analysis and find a highly prevalent, CVD-protective pattern in the population.Alas, another study has found an unhealthy dietary pattern, this time in UK adults, is prospectively linked with adverse CVD outcomes [3]. The persistent high prevalence of poor diets is unacceptable. We never would have accepted a persistent smoking prevalence of 46% [10]. We acted by adopting a comprehensive suite of policies to tackle tobacco, including industry regulations, and as a result, the prevalence of smoking today is just 14% [10]. We need a whole-diet strategy, not one that relies on tinkering with a couple of nutrients. And we need to start asking more people what they eat on a regular basis. There is hope we can tackle poor diets and obesity, but we are not going to get there with more of the same old strategies.

## Data Availability

Not applicable
